# Short- and long-term outcomes of single-port versus multiport laparoscopic radical gastrectomy for gastric cancer: a meta-analysis of propensity score-matched studies and randomized controlled trials

**DOI:** 10.1186/s12893-023-02134-z

**Published:** 2023-08-09

**Authors:** Guangxu Zhu, Xiaomin Lang, Shengjie Zhou, Bowen Li, Qihang Sun, Lei Yu, Qingshun Zhu, Wei Lang, Xuguang Jiao, Shengyong Zhai, Jinqiu Xiong, Yanan Fu, Jianjun Qu

**Affiliations:** 1 Department of General surgery, Weifang People’s Hospital, Weifang, Shandong China; 2 Department of Anesthesiology, Weifang People’s Hospital, Weifang, Shandong China; 3 Weifang Medical University, Weifang, Shandong China; 4 Medical administration dept, Weifang People’s Hospital, Weifang, Shandong China

**Keywords:** Gastric cancer, Gastrectomy, Single-port laparoscopic gastrectomy, Multiport laparoscopic gastrectomy, Short-term outcomes, Long-term outcomes

## Abstract

**Background:**

At present, there is no convincing evidence-based medical basis for the efficacy of single-port laparoscopic gastrectomy. To make a high-quality comparison of the short- and long-term outcomes of single-port laparoscopic gastrectomy versus multiport laparoscopic gastrectomy, we performed this meta-analysis, which only included propensity score-matched studies and randomized controlled trials comparing single-port laparoscopic gastrectomy with multiport laparoscopic gastrectomy for patients with gastric cancer.

**Methods:**

Data were retrieved from the electronic databases PubMed, EMBASE, Medline, Cochrane Library, CNKI, Wanfang and VIP up to January 2023, and the data included the outcomes of treatment after single-port laparoscopic gastrectomy and multiport laparoscopic gastrectomy. The primary outcomes were early complications, survival rate after surgery at 1 year, and survival rate after surgery at 5 years. The secondary outcomes were number of pain medications, mean operation time, estimated blood loss, hospital mortality, time to first soft fluid diet, time to first flatus, hospital stay after surgery, and retrieved number of lymph nodes. The Jadad score and Newcastle‒Ottawa scale were used to assess the quality of the included studies.

**Results:**

After screening, 9 studies were finally included, including 988 patients. The meta-analysis results showed that estimated blood loss (MD=-29.35, 95% CI: -42.95-15.75, P < 0.0001), hospital stay (MD=-0.99, 95% CI:-1.82~-0.17, P = 0.02), and number of pain medications(MD=-0.65, 95% CI:-1.07~-0.23, P = 0.002) in the single-port laparoscopic gastrectomy group were better than those in the multiport laparoscopic gastrectomy group. There is no significant difference between the single-port laparoscopic gastrectomy group and the multiport laparoscopic gastrectomy group in mean operation time(MD = 5.23,95% CI:-16.58~27.04,P = 0.64), time to first soft fluid diet(MD=-0.06,95% CI: -0.30~0.18,P = 0.63), time to first flatus(MD=-0.18,95% CI:-0.43~0.07,P = 0.16), early complication(OR = 0.73,95% CI:0.50~1.09,P = 0.12), hospital mortality(OR = 1.00,95% CI:0.09~11.16,P = 1.00), retrieved number of lymph nodes(MD=-1.15, 95% CI:-2.71~0.40, P = 0.15), survival rate after surgery 1 year(OR = 2.14,95% CI:0.50~9.07,P = 0.30), and survival rate after surgery 5 year(93.7 vs. 87.6%; p = 0.689).

**Conclusion:**

This meta-analysis showed that single-port laparoscopic gastrectomy is both safe and feasible for laparoscopic radical gastrectomy for gastric cancer, with similar operation times and better short-term outcomes than multiport laparoscopic gastrectomy in terms of hospital stay, postoperative pain, and estimated blood loss. There was no significant difference in long-term outcomes between single-port laparoscopic gastrectomy and multiport laparoscopic gastrectomy.

## Introduction

In 1994, Kitano et al. [10]. first reported radical laparoscopic gastrectomy, which has been widely used by clinicians at home and abroad due to its minimally invasive nature and adequate oncologic outcome. In recent years, with the continuous progress of laparoscopic surgical techniques and the continuous efforts of surgeons, single-port laparoscopic radical gastrectomy for gastric cancer has gradually emerged and become a topic of interest. That is, the operation is completed with a small incision of 3 ~ 4 cm in the umbilical part so that patients have less damage to the integrity of the abdominal wall, less postoperative pain and get out of bed earlier, recover faster, have fewer complications related to postoperative incision, and have a shortened length of hospital stay. Although clinical studies on single-port laparoscopic surgery for gastric cancer have been reported at home and abroad, the number of cases is relatively small, and there are some differences in the research results [1–3]. Therefore, the meta-analysis method was adopted in this study to systematically analyze the short-term and long-term efficacy of laparoscopic single-port gastrectomy and multiport laparoscopic gastrectomy in the treatment of gastric cancer to provide a reference and basis for the clinical treatment of gastric cancer.

## Methods

This meta-analysis of propensity score-matched studies and randomized controlled trials was based on the reporting items for systematic study reviews and meta-analyses statements (PRISMA) [11].

### Literature retrieval

English literature was retrieved from PubMed, EMBASE, Medline, and Cochrane Library. Chinese literature was searched in the CNKI, VIP, and Wanfang databases. The search keywords were ((“stomach” OR “gastric”) AND (“cancer” OR “tumor” OR “carcinoma” OR “neoplasm”) AND (“single port” OR “single incision”) AND (“laparoscopic” OR “laparoscope”). The search ended in 2023.01.

### Inclusion and exclusion criteria

The inclusion criteria were as follows: (1) the types of gastric surgery were distal gastrectomy or total gastrectomy; (2) the study was either a propensity score-matched study (PSMs) or a randomized controlled trial study (RCT); (3) the study compared short- and long-term outcomes of the single-port laparoscopic gastrectomy (SLG) group and multiport laparoscopic gastrectomy (MLG) group after distal gastrectomy or total gastrectomy; and (4) the study provided data on any short- and long-term outcomes. The exclusion criteria were as follows: (1) literature that does not contain data from both SLG and MLG comparative studies; (2) incomplete data or data that cannot be extracted and applied; (3) repetitive literature; (4) reviews, case reports, conference abstracts, and other noncontrolled studies.

### Data extraction and quality assessment

Literature screening and data extraction were completed independently by two researchers and cross-checked. If inconsistent results or differences were found during the audit, they were resolved through discussion via assistance by the third investigator to determine whether they would be included in the study. Literature screening was performed by reading the title, abstract and full text of the paper.

The extracted data included the basic information included in the study, baseline characteristics of the subjects, intervention measures and outcome indicators. Basic information included in the study includes the research topic, the name of the first author, the country, the publication of journals and periodicals, and the year of publication. Baseline characteristics included sample size, age, digestive tract reconstruction, and other indicators. The primary outcomes were early complications, survival rate after surgery at 1 year, and survival rate after surgery at 5 years. The secondary outcomes were number of pain medications, mean operation time, estimated blood loss, hospital mortality, time to first soft fluid diet, time to first flatus, hospital stay after surgery, and retrieved number of lymph nodes. The RCT studies were evaluated using the Jadad Modified Scale [12]. The Newcastle‒Ottawa (NOS) literature quality evaluation scale was used to evaluate nRCT research [13].

### Statistical analysis

ReviewManager5.4 software was used for the meta-analysis of the final included data, and the analysis results were presented in the form of a forest map. Odds ratio (OR) analysis was used for counting data (early complications, hospital mortality and pain control number, etc.). Standard mean difference (MD) analysis was used for measurement data (mean operating time, estimated blood loss, time to first soft fluid diet, etc. ), and 95% CI was used for interval assessment. I^2^ was used for quantitative analysis of heterogeneity. When I^2^ was greater than 50%, it indicated statistical heterogeneity in the included studies. Sources of heterogeneity need to be analyzed, and subgroup analysis or random effects models should be used to analyze factors that may lead to heterogeneity. P < 0.05 indicated that the difference between the two groups was statistically significant.

## Results

### Inclusion of literature and its quality assessment

**Fig. 1:**
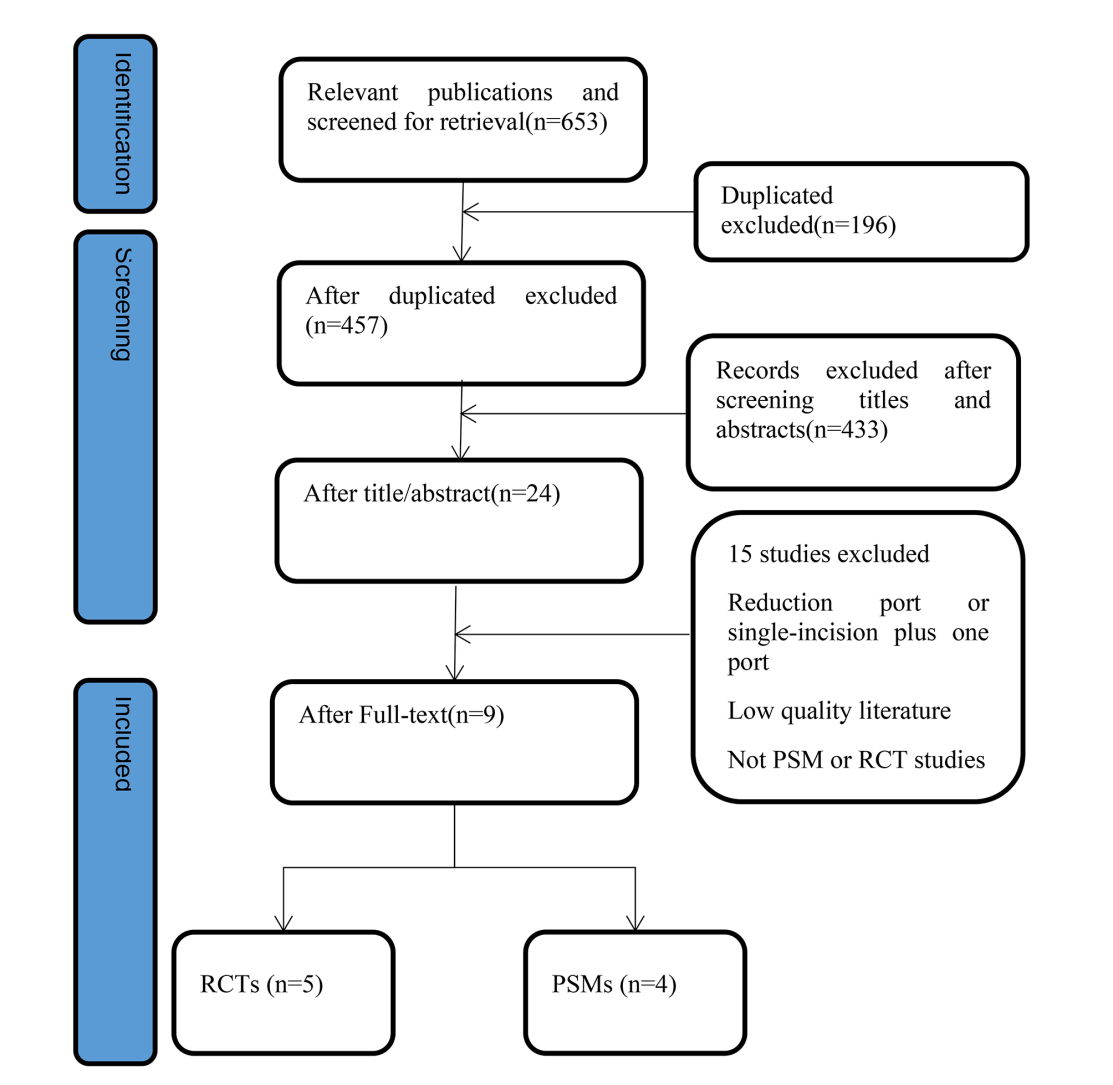
Flow chart of literature search process

We initially obtained 653 papers through English and Chinese databases and finally included 9 papers through literature screening by the inclusion and exclusion criteria(Fig. 1). Among the literature included, 5 papers were RCT studies, and 4 were PSM studies. The results of the literature quality assessment are shown in Table 1.

**Table 1:** Characteristics of studies included

Study	Year	Country	Interventions		Operative method		Age		BMI		Surgical outcome	
			SLG group	MLG group	SLG group	MLG group	SLG group	MLG group	SLG group	MLG group		
PSMs(n = 4)												NOS
M D Zang [1]	2022	China	14	14	DG	DG	< 60,10(10/14);>60,4(4/14)	< 60,11(11/14);>60,3(3/14)	20.8 ± 0.8	22.9 ± 0.4	①②③④⑤⑥⑦⑧⑨	7
Boram Lee [2]	2021	Korea	99	198	DG	DG	58.3 ± 12.2	60.7 ± 11.8	23.3 ± 2.95	24.6 ± 3.28	① ② ③④⑤⑥⑦⑧⑨	8
Omori T [3]	2016	Japan	50	50	DG + TG	DG + TG	64.5 ± 11.2	64.4 ± 9.7	22.0 ± 3.2	22.7 ± 3.4	① ② ③④⑤⑥⑦⑧⑨⑩	7
Sang-Hoon Ahn [4]	2014	Korea	50	50	DG	DG	60.7 ± 11.9	58.2 ± 12.5	23.0 ± 3.3	24.1 ± 3.1	① ② ③④⑤⑥⑦⑧⑨	6
RCTs(n = 5)												Jadad scale
So Hyun Kang [5]	2022	Korea	43	40	DG	DG	62.0 ± 9.7	58.9 ± 13.0	24.3 ± 2.8	24.4 ± 3.6	① ② ③④⑤⑥⑧⑨	5
Yantong Yang [6]	2022	China	48	47	DG + TG	DG + TG	60.58 ± 6.19	60.37 ± 6.28	NA	NA	① ② ⑤⑥⑧⑨⑩	6
Omori T [7]	2020	Japan	50	51	DG	DG	64.7 ± 1.4	63.9 ± 1.4	22.1 ± 0.4	22.7 ± 0.4	① ② ③④⑤⑦⑧⑨⑩	6
Kaining Zhu [8]	2020	China	42	44	DG	DG	61.38 ± 11.57	60.86 ± 10.57	21.64 ± 2.27	21.66 ± 2.74	① ② ③⑤⑥⑧	6
Bo Yang [9]	2020	China	49	49	DG + TG	DG + TG	53.26 ± 4.11	54.21 ± 5.03	22.36 ± 1.02	22.20 ± 1.11	① ② ③⑤⑥⑧	5

①Mean operation time ②Estimated blood loss ③Retrieved number of lymph nodes ④Time to first soft fuid diet

⑤Time to first flatus ⑥Hospital stay ⑦Number of pain control ⑧Early complication ⑨Mortality ⑩Longterm outcomes

NA Not Available PSMs Propensity score-matched studies RCTs Randomized controlled trials studies BMI Body mass index SLG single-port laparoscopic gastrectomy MLG multi-port laparoscopic gastrectomy DG Distal gastrectomy TG Total gastrectomy

### Results of the meta-analysis of the primary outcomes

**Fig. 2:**
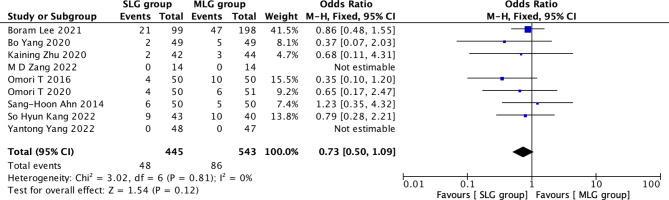
Forest plots of comparision between SLG and MLG on early complication

#### Early complications

A total of 988 patients in 9 papers were meta-analyzed in terms of early complications [1–9]. The results from the combined literature showed that SLG did not have a significant advantage over MLG in early complications (OR = 0.73, 95% CI: 0.50~1.09, P = 0.12). Heterogeneity test (P = 0.81, I^2^ = 0%). (Fig. 2).

**Fig. 3:**
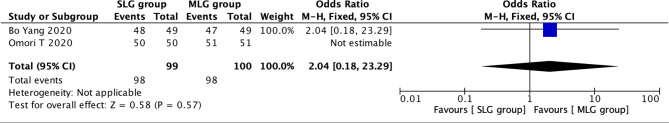
Forest plots of comparision between SLG and MLG on long-term outcomes

#### Long-term outcomes

A total of 199 patients in 2 papers were meta-analyzed in terms of survival rate after surgery at 1 year [6, 7]. The results from the combined literature showed that SLG did not have a significant advantage over MLG in long-term outcomes (OR = 2.14, 95% CI: 0.50~9.07, P = 0.30). (Fig. 3). Omori T [3] reported a 5-year survival rate after surgery with no statistically significant difference between the two groups (93.7 vs. 87.6%; p = 0.689).

### Results of the meta-analysis of the secondary outcomes

**Fig. 4:**
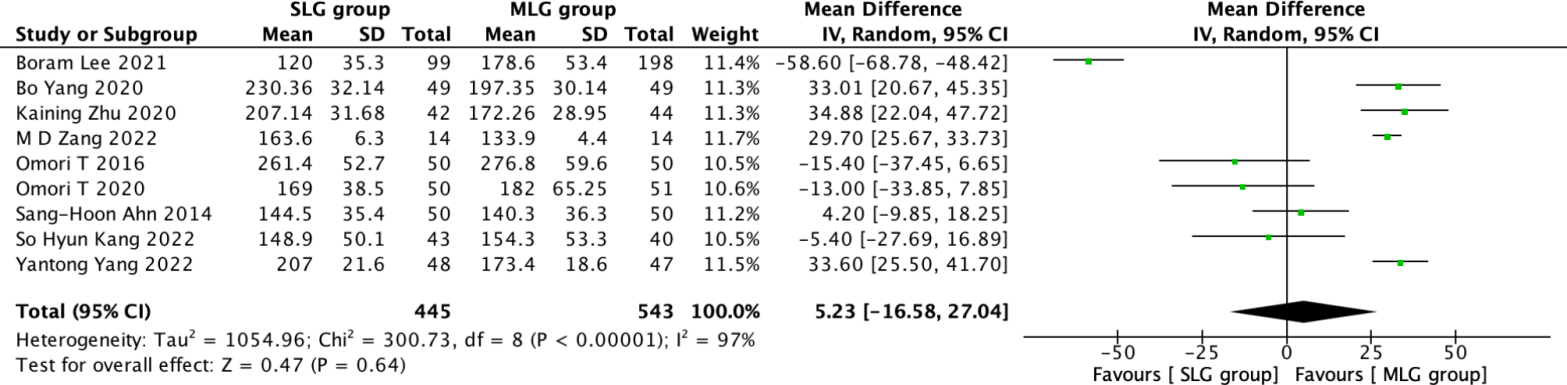
Forest plots of comparision between SLG and MLG on mean operation time

#### Mean operation time

A total of 988 patients in 9 papers were meta-analyzed in terms of mean operating time [1–9]. The results from the combined literature showed that SLG did not have a significant advantage over MLG in mean operating time. (MD = 5.23, 95% CI: -16.58~27.04, P = 0.64). Heterogeneity test (P < 0.00001, I^2^ = 97%). (Fig. 4).

**Fig. 5:**
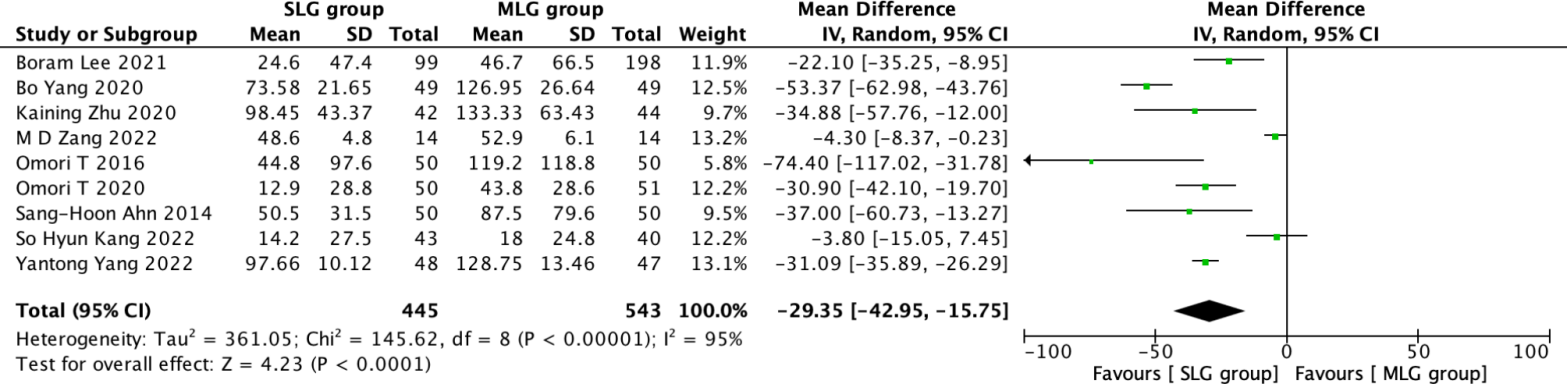
Forest plots of comparision between SLG and MLG on estimated blood loss

#### Estimated blood loss

A total of 988 patients in 9 papers were meta-analyzed in terms of estimated blood loss [1–9]. The results from the combined literature showed that SLG had a significant advantage over MLG in estimated blood loss (MD=-29.35, 95% CI: -42.95~-15.75, P < 0.0001). Heterogeneity test (P < 0.^00001, I2^ = 95%). (Fig. 5).

**Fig. 6:**
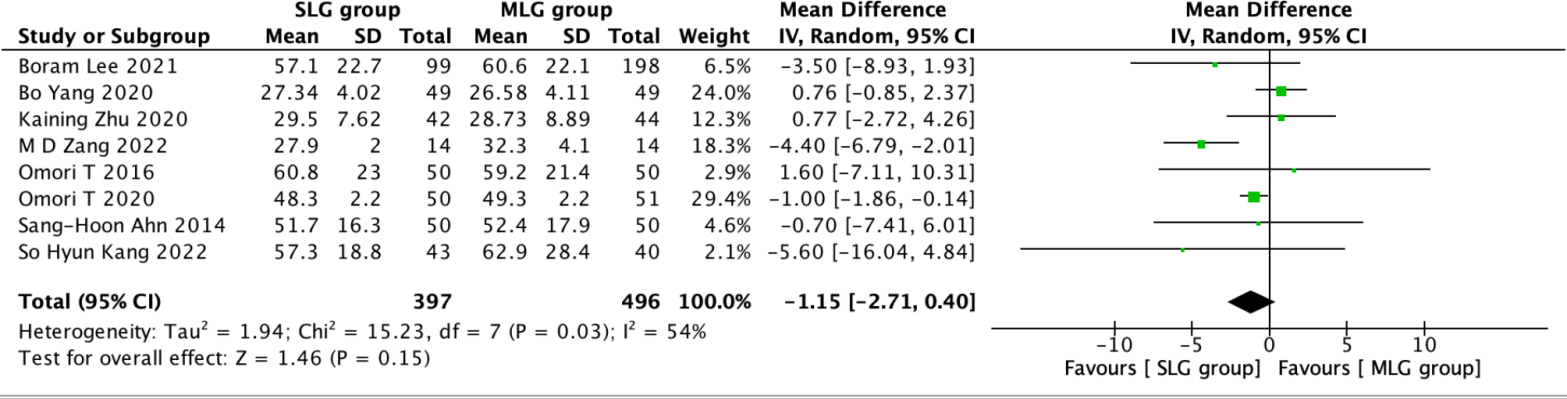
Forest plots of comparision between SLG and MLG on retrieved number of lymph nodes

#### Retrieved number of lymph nodes

A total of 893 patients in 8 papers were meta-analyzed in terms of the retrieved number of lymph nodes [1–5, 7–9]. The results from the combined literature showed that SLG did not have a significant advantage over MLG in the retrieved number of lymph nodes. (MD=-1.15, 95% CI:-2.71~0.40, P = 0.15). Heterogeneity test (P = 0.03, I^2^ = 54%). (Fig. 6).

**Fig. 7:**
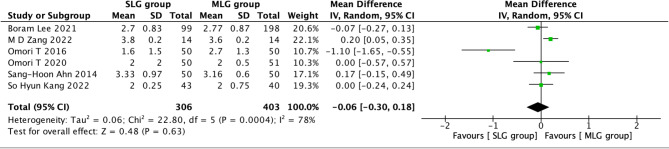
Forest plots of comparision between SLG and MLG on time to first soft fluid diet

#### Time to first soft fluid diet

A total of 709 patients in 6 papers were meta-analyzed in terms of time to first soft fluid diet [1–5 ,7]. The results from the combined literature showed that SLG did not have a significant advantage over MLG in time to first soft fluid diet (MD=-0.06, 95% CI: -0.30~0.18, P = 0.63). Heterogeneity test (P = 0.0004, I^2^ = 78%). (Fig. 7).

**Fig. 8:**
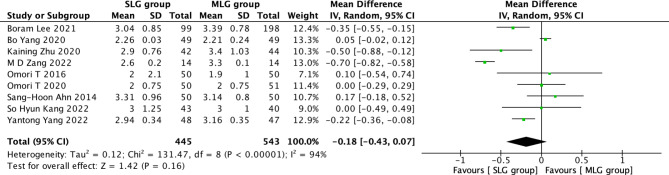
Forest plots of comparision between SLG and MLG on time to first flatus

#### Time to first flatus

A total of 988 patients in 9 papers were meta-analyzed in terms of time to time to first flatus [1–9]. The results from the combined literature showed that SLG did not have a significant advantage over MLG in time to first flatus (MD=-0.18, 95% CI:-0.43~0.07, P = 0.16). Heterogeneity test (P < 0.00001, I^2^ = 94%). (Fig. 8).

**Fig. 9:**
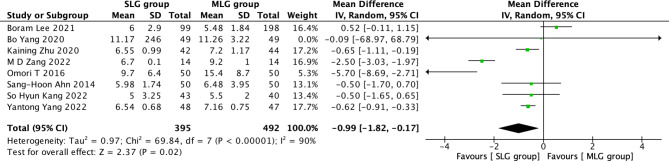
Forest plots of comparision between SLG and MLG on hospital stay

#### Hospital stay

A total of 887 patients in 8 papers were meta-analyzed in terms of hospital stay after surgery [1–9]. The results from the combined literature showed that SLG had a significant advantage over MLG in hospital stay after surgery (MD=-0.99, 95% CI:-1.82~-0.17, P = 0.02). Heterogeneity test (P < 0.^00001, I2^ = 90%). (Fig. 9).

**Fig. 10:**
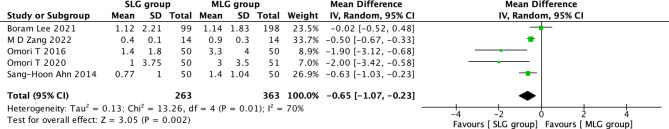
Forest plots of comparision between SLG and MLG on number of pain medications

#### Number of pain medications

A total of 626 patients in 5 papers were meta-analyzed in terms of pain medications [1–4, 7]. The results from the combined literature showed that SLG had a significant advantage over MLG in pain medications (MD=-0.65, 95% CI: -1.07~-0.23, P = 0.002). Heterogeneity test (P = 0.^01, I2^ = 70%). (Fig. 10).

**Fig. 11:**
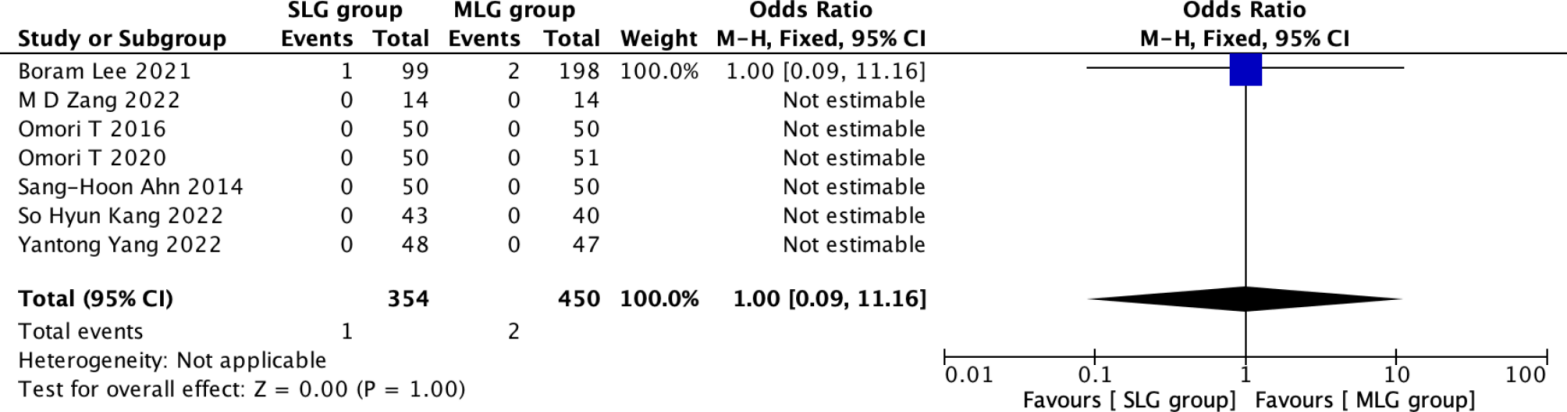
Forest plots of comparision between SLG and MLG on mortality

#### Mortality

A total of 804 patients in 7 papers were meta-analyzed in terms of mortality [1–7]. The results from the combined literature showed that SLG did not have a significant advantage over MLG in mortality (OR = 1.00, 95% CI: 0.09~11.16, P = 1.00). (Fig. 11).

### Sensitivity analysis

The results of this study show that there is high heterogeneity among mean operation time, estimated blood loss, retrieved number of lymph nodes, time to first soft fluid diet, time to first flatus, hospital stay, and number of pain controls. Therefore, we excluded the studies one by one and then analyzed the sensitivity of the literature. The results show that the combination of estimated blood loss, retrieved number of lymph nodes, time to first soft fluid diet, time to first flatus, and number of pain control in this study is basically reliable. However, the results of mean operation time and hospital stay were not stable. The reasons may be related to potential selection bias (such as differences in the patient inclusion criteria and exclusion criteria, tumor staging, surgical methods, etc.), surgical level of the surgeon, and medical device specifications.

### Publication bias analysis

**Fig. 12:**
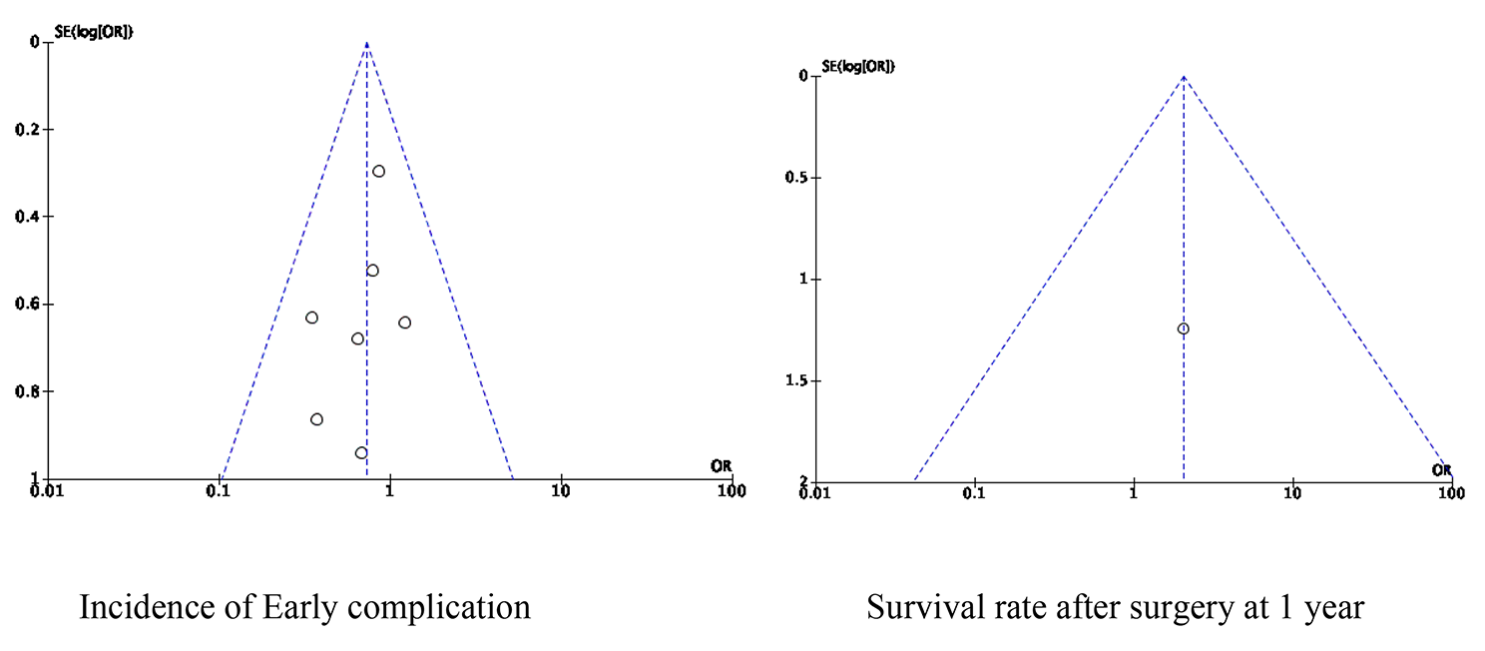
Funnel chart with incidence of Early complication and Survival rate after surgery at 1 year

With the incidence of early complications and survival rate after surgery as the main outcome indices, a funnel chart was made to assess whether there was bias in this study. The results show that the scatter points are symmetrically distributed in the funnel chart, and only one item is distributed outside the funnel chart, indicating that the likelihood of publication bias in this meta-analysis is low (Fig. 12).

## Discussion

Due to the high technical difficulty and difficult lymph node dissection of SLG, experts at home and abroad dispute the safety of the operation and the effect of radical oncology. Therefore, we conducted a systematic review that included prospective randomized controlled trials and matched analysis studies to analyze the short- and long-term efficacy of SLG.

In this study, all the papers analyzed were from Southeast Asia (China, Japan, Korea, etc.). According to the relevant research results, gastric cancer ranks fifth in the global incidence rate and is mainly found in East Asian countries [14]. According to statistics, in 2020, an estimated 769,000 people worldwide died of gastric cancer, with at least 1 million new cases of gastric cancer [14]. In this study, early postoperative complications and mortality in both groups were summarized and analyzed. The results showed no significant difference between the two groups, indicating that SLG is safe and feasible. According to the traditional view, the operating time of SLG is longer than that of MLG due to the limited operating space and lack of exposure to the auxiliary field, which leads to a longer operating time [15, 16]. However, the results of this study showed no significant difference in operating time between the SLG and MLG groups (MD = 5.23, 95% CI: -16.58 ~ 27.04, P = 0.64). The author believes that the reasons why SLG does not increase the operating time include the following: (1) these surgeons have rich surgical experience and have undergone a long learning curve. According to Kang et al., the SLG learning curve was 30 cases, and the mean operating time after reaching the learning curve was 118 ± 34.5 min, which was equal to or faster than that of MLG [17]. During the implementation of SLG technology in gastric cancer, the requirements for the surgical team are extremely high. Due to the lack of a triangular traction plane, there are significant difficulties in the dissection of lymph nodes and reconstruction of the digestive tract in gastric cancer. At the same time, SLG technology also has high requirements for assistive hands. Since the lens, left hand grip forceps, and right hand energy instruments all enter the abdominal cavity through a single-port puncture device, the surgeon’s operation may cause laparoscopic shaking, resulting in unstable display images. Therefore, the person holding the laparoscope needs to firmly support it with both hands, maintain the stability of the lens, flexibly adjust the lens angle, avoid the surgeon’s instruments, prevent collisions, and keep the surgeon’s instruments above the front of the laparoscope. The difficulty of SLG surgery is significant and requires collaboration with a well-trained team, so it takes a long time for surgeons to master this technique. Surgeons should gradually transition from simple surgery to complex surgery. If they encounter difficulties, they should quickly change the surgical method and switch to porous or open surgery to ensure maximum benefit for patients. (2) SLG has only one incision around the umbilical cord, and there is no need to add or prolong the incision. However, MLG often requires five abdominal incisions [3]. (3) The whole operation is performed by the surgeon alone to avoid visual field tremor, incorrect traction and tissue injury caused by the assistant [8]. The results of this study showed that intraoperative bleeding in the SLG group was lower than that in the MLG group. The reasons for less bleeding during SLG include [8]: (1) the operation is mainly performed by the surgeon alone, which can reduce bleeding caused by an auxiliary error; (2) in SLG, only ultrasonic knives can be used to bite slowly in a small area to deal with the anatomical plane more finely; and (3) SLG can reduce the risk of bleeding caused by trocar holes.

In terms of postoperative recovery, the SLG postoperative pain control time and hospital stay were shorter than those of the MLG, and the difference was statistically significant (all P < 0.05). SLG includes an incision around the umbilical cord that does not need to be added or prolonged, which minimizes damage to the abdominal wall and significantly reduces postoperative pain and discomfort for patients. The length of the incision is smaller, reducing postoperative intestinal paralysis caused by the use of analgesics, so the time to get out of bed is short, accelerating postoperative recovery and shortening hospitalization time. However, the surgical procedures, lymph node dissection, and intestinal traction were not significantly reduced in the SLG group, so there was no significant difference in time to first soft fluid diet and time to first flatus between the two groups.

The number of lymph node dissections is the key factor in determining the degree of radical resection and long-term prognosis of gastric cancer. D1 + or D2 lymph node dissection must be performed for early gastric cancer, and D2 lymph node dissection must be performed for advanced gastric cancer [18]. Celiac lymph node dissection is the key and difficult point of radical laparoscopic resection of gastric cancer, especially in single-port radical laparoscopic resection of gastric cancer. The results of this study showed that there was no significant difference in the number of lymph node dissections between the SLG and MLG groups. According to the relevant literature, in laparoscopic radical resection of distal gastric cancer, there are some difficulties in lymph node dissection in the central region of the upper margin of the pancreas, and some skills and strategies need to be adopted. With the development of laparoscopic instruments such as soft endoscopes, 3D laparoscopes, flexible grips and rotary cutting occluders, the efficiency of lymph node dissection will be effectively improved, and SLG has been further promoted and applied in gastric cancer surgery [19, 20]. The results of this study showed that there was no significant difference in the 1- or 5-year overall survival rates between the two groups [3, 6–7]. Therefore, SLG is feasible in the radical resection of gastric cancer.

Compared to SLG’s auxiliary incision, MLG’s “single incision” can be used not only for surgical operations but also for specimen collection, and the incision is more fully utilized. Combined with relevant literature reports, we believe that the indications for radical gastrectomy by laparoscopic single port surgery are [1]: (1) early gastric cancer; (2) no history of abdominal surgery; (3) no abdominal adhesion; and (4) body mass index < 25 kg/m^2^. All patients in this meta-analysis had BMI < 25. Obese individuals (BMI > 25) have a narrow abdominal space and a hypertrophic omentum, which makes SLG surgery difficult to perform and expose, increasing the difficulty of surgery. (5) Another indication is if there are special requirements for cosmetics.

There are still some shortcomings in this meta-analysis, including (1) the sample size of the prospective randomized controlled studies is relatively small; (2) the literature quality is low; (3) the design defect of the PSM study itself; (4) only Chinese and English literature was included (5) Differences in tumor stage, range of lymph node dissection, and surgeon level in different research centers may lead to significant heterogeneity among studies. (6) This meta-analysis did not include a separate subgroup analysis of laparoscopic distal gastrectomy and total gastrectomy. At a later stage, with the development of a series of studies on single-port laparoscopic total gastrectomy, we will conduct more detailed research based on the types of gastrectomy.

In summary, SLG is both safe and feasible for laparoscopic radical gastrectomy for gastric cancer, with similar operation times and better short-term outcomes than MDG in terms of hospital stay, postoperative pain, and estimated blood loss. There was no significant difference in long-term outcomes between SLG and MLG.

## Data Availability

All data generated or analysed during this study are included in this published article.

## References

[1] Zang MD, Chen J, Zhang Y (2022). Analysis on perioperative safety and feasibility of pure single-port laparoscopic distal gastrectomy for gastric cancer.Chin. J Gastrointest Surg.

[2] Lee BLeeSangIYounKanghaeng (2021). Comparing the short-term outcomes and cost between solo single-incision distal gastrectomy and conventional multiport totally laparoscopic distal gastrectomy for early gastric cancer: a propensity score-matched analysis. Ann Surg Treat Res.

[3] Omori T, Fujiwara Y, Moon J (2016). Comparison of single-incision and conventional multi-port laparoscopic distal gastrectomy with D2Lymph node dissection for gastric Cancer: a propensity score-matched analysis. Ann Surg Oncol.

[4] Sang-Hoon Ahn,Sang-Yong Son,do Hyun Jung,et al.Pure single-port laparoscopic distal gastrectomy for early gastric cancer: comparative study with multi-port laparoscopic distal gastrectomy.J Am Coll Surg. 2014, 219(5):933–43. 10.1016/j.jamcollsurg.2014.07.009. 10.1016/j.jamcollsurg.2014.07.009 25256369

[5] So Hyun Kang,Mira Yoo,Duyeong Hwang,et al.Postoperative pain and quality of life after single-incision distal gastrectomy versus multiport laparoscopic distal gastrectomy for early gastric cancer - a randomized controlled trial.2023, 37(3):2095–103. 10.1007/s00464-022-09709-6. 10.1007/s00464-022-09709-6 PMC9616415 36307602

[6] Yantong YANG, Pengke ZHI, Bo ZHOU et al. Comparison of single-incision and conventional multi-incision laparoscopic radical gastrectomy in treatment of early gastric Cancer Huaihai Med 2022,40(3):221–5.DOI: 10.14126 /j.cnki.1008–7044.

[7] Takeshi Omori,Kazuyoshi Yamamoto,Hisashi, Hara et al. A randomized controlled trial of single-port versus multi-port laparoscopic distal gastrectomy for gastric cancer.Surg Endosc. 2021,35(8):4485–4493. 10.1007/s00464-020-07955-0. 10.1007/s00464-020-07955-0 32886237

[8] Kai-ning ZHU, Yi CAO, Zong-feng FENG et al. A prospective controlled study of short-term efficacy and quality of life of transumbilical single-port laparoscopy and conventional five-port laparoscopic distal gastrectomy.Journal of laparoscopic surgery.2020,25(1):35–41.DOI: 10.13499 /j.cnki.fqjwkzz.

[9] YANG Bo. Radical gastrectomy and traditional five-port laparoscopic D2 radical gastrectomy for patients with early gastric cancer.Medical J Chin People’s Health 2020,32(22):115–7.doi: 10. 3969 / j. issn. 1672 – 0369.

[10] Kitano S, Iso Y, Moriyama M et al. Laparoscopy-assisted Billroth I gastrectomy[J]. Surg Laparosc Endosc 1994,4(2):146–8. 8180768

[11] Liberati A, Altman DG, Tetzlaff J (2009). The PRISMA statement for reporting systematic reviews and meta-analyses of studies that evaluate healthcare interventions: explanation and elaboration. BMJ.

[12] Jadad AR, Moore RA, Carroll D (1996). Assessing the quality of reports of randomized clinical trials: is blinding necessary?. Control Clin Trials.

[13] Luchini C, Stubbs B, Solmi M (2017). Assessing the quality of studies in Meta-analyses: advantages and limitations of the Newcastle Ottawa Scale. World J Meta-Anal.

[14] Sung H, Ferlay J, Siegel RL (2021). Global Cancer Statistics 2020:GLOBOCAN estimates of incidence and Mortality Worldwide for 36 cancers in 185 Countries[J]. CA Cancer J Clin.

[15] Junfeng Z (2021). Sheng Lin,Sida Sun,et al.Effect of single-incision laparoscopic distal gastrectomy guided by ERAS and the influence on immune function. World J Surg Oncol.

[16] Guang-Sheng Du,En-Lai Jiang,Yuan Qiu,et al.Single-incision plus one-port laparoscopic gastrectomy versus conventional multi-port laparoscopy-assisted gastrectomy for gastric cancer: a retrospective study. Surg Endosc. 2022, 36(5):3298–307. 10.1007/s00464-021-08643-3. 10.1007/s00464-021-08643-3 34313862

[17] So Hyun Kang,Yo-Seok Cho,Sa-Hong Min,et al.Early experience and learning curve of solo single-incision distal gastrectomy for gastric cancer: a review of consecutive 100 cases.Surg Endosc. 2019, 33(10):3412–8. 10.1007/s00464-018-06638-1. 10.1007/s00464-018-06638-1 30604257

[18] Kim SM, Ha MH, Seo JE (2016). Comparison of single-port and reducedport totally laparoscopic distal gastrectomy for patients with early gastric cancer. Surg Endosc.

[19] Lee Y, Kim HH. Single-incision laparoscopic gastrectomy for gastric Cancer.J gastric Cancer. 2017, 17(3):193–203. 10.5230/jgc. 10.5230/jgc.2017.17.e29 PMC5620088 28970949

[20] So Hyun Kang,Yongjoon Won,Kanghaeng Lee,et al.Three-dimensional (3D) visualization provides better outcome than two-dimensional (2D) visualization in single-port laparoscopic distal gastrectomy: a propensity-matched analysis. Langenbecks Arch Surg. 2021;406(2):473–8. 10.1007/s00423-020-01952-6. 10.1007/s00423-020-01952-6 32748044

